# Human Lung Mast Cells Impair Corticosteroid Responsiveness in Human Airway Smooth Muscle Cells

**DOI:** 10.3389/falgy.2021.785100

**Published:** 2021-12-29

**Authors:** Abdulrahman Alzahrani, Jameel Hakeem, Michael Biddle, Fahad Alhadian, Aamir Hussain, Latifa Khalfaoui, Katy M. Roach, Omar Tliba, Peter Bradding, Yassine Amrani

**Affiliations:** ^1^Department of Respiratory Sciences, Clinical Sciences, University of Leicester, Glenfield Hospital, Leicester, United Kingdom; ^2^Department of Applied Medical Sciences, Applied College, Albaha University, Albaha, Saudi Arabia; ^3^Department of Biomedical Sciences, College of Veterinary Medicine, Long Island University, Brookville, NY, United States

**Keywords:** severe asthma, corticosteroid resistance, mast cell (MC), growth factors, gene array

## Abstract

The mechanisms underlying corticosteroid insensitivity in severe asthma have not been elucidated although some indirect clinical evidence points toward a role of mast cells. Here, we tested the hypothesis that mast cells can drive corticosteroid insensitivity in airway smooth muscle cells, a key player in asthma pathogenesis. Conditioned media from resting or FcεR1-activated human lung mast cells were incubated with serum-deprived ASM cells (1:4 dilution, 24 h) to determine their impact on the anti-inflammatory action of fluticasone on ASM cell chemokine expression induced by TNFα (10 ng/ml). Conditioned media from FcεR1-activated mast cells (but not that from non-activated mast cells or control media) significantly reduced the ability of 100 nM fluticasone to suppress ASM TNFα-dependent CCL5 and CXCL10 production at both mRNA and protein levels. In contrast, fluticasone inhibition of CXCL-8 production by TNFα was still preserved in the presence of activated mast cell conditioned media. Transcriptomic analysis validated by individual qPCR assays revealed that activated mast cell conditioned media dramatically reduced the number of anti-inflammatory genes induced by fluticasone in ASM cells. Our study demonstrates for the first time that conditioned media from FcεR1-activated mast cells blunt the anti-inflammatory action of corticosteroids in ASM cells by altering their transactivation properties. Because infiltration of mast cells within the ASM bundles is a defining feature of asthma, mast cell-derived mediators may contribute to the glucocorticoid insensitivity present in severe asthma.

## Introduction

Both preclinical and clinical studies indicate a central role for mast cells in the pathogenesis of asthma, through their unique ability to produce an array of mediators capable of regulating key features of both the innate and adaptive immunity in the lungs (1). Activation of mast cells by both allergic and non-allergenic stimuli has been traditionally linked to the initiation and perpetuation of the allergic inflammation cycle via the secretion of different Type 2 (or Th2) cytokines. Interleukin 4 (IL-4) and IL-13 regulate Th2 cell proliferation and B cell production of allergen-specific IgE, while IL-5 drives eosinophilic inflammation, all key features of the allergic responses in asthma. Other mast cell mediators also contribute to key structural/clinical features of asthma such as mucus hypersecretion, epithelium permeability, airway hyper-responsiveness (AHR), bronchoconstriction and airway remodeling (1). Multiple triggers contribute to mast cell activation in asthma including stimulation of the high affinity IgE receptor FcεRI by allergens, ligands of the Toll like receptors, and cytokines activating the alarmin receptors (TSLP, IL-33) (1). These different triggers, likely acting in concert, lead to both the acute and chronic mast cell activation that is observed in severe asthma, an important feature that is present irrespective of the clinical phenotype (2).

Asthma is also characterized by the presence of mast cells within different compartments of the airways. Infiltration of mast cells has been described within the epithelium, submucosa layer and airway smooth muscle and has been shown to correlate with disease severity [reviewed in (3)]. Bidirectional interactions between human lung mast cells, and structural airway tissues have been demonstrated using a co-culture model system with mast cells being able to drive pro-asthmatic responses in airway smooth muscle (ASM) cells including loss of β2-adrenoceptor function indirectly via the paracrine action of secreted TGFβ (4), or following direct cell-cell physical interaction (5, 6). Similarly, β2-adrenoceptor dysfunction can also be observed in human lung mast cells as a result of the autocrine action of secreted SCF (5, 7). These different studies show that activation of mast cells within the airways can alter the therapeutic response of lung structural cells to current anti-asthma therapies.

In this study, we tested whether mast cells could also alter the therapeutic response to glucocorticoids (GCs). This hypothesis is part supported by studies showing a marked reduction in the use of both inhaled and oral GCs in severe allergic asthmatics following treatment with omalizumab therapy, an anti-IgE monoclonal antibody (8–10). MacDonald and colleagues, when looking at the overall clinical impact of omalizumab of 42 clinical studies, confirmed that omalizumab treatment for >2 months or longer led patients to either reduce or stop their usage of inhaled/oral GCs (11). How mast cells regulate patients' response to GC therapy remains unknown but the possibility that mast cell mediators directly or indirectly modulate GC sensitivity of lung structural cells is an interesting hypothesis. Here we provide compelling evidence that mediators released by activated mast cells can contribute to disease severity by blunting the therapeutic response of GCs in human ASM cells via mechanisms involving a reduced GRα transactivation (i.e., expression of anti-inflammatory genes).

## Materials and Methods

### Study Participants

All participants gave written informed consent, and the study was approved by the Leicestershire, Northamptonshire, and Rutland Research Ethics Committee (references: 4977, 04/Q2502/74 and 08/H0406/189).

### Culture of Human Airway Smooth Muscle Cells

Primary human ASM cells were isolated from endobronchial biopsies as previously described (12).

### Mast Cell Isolation, Culture and Stimulation

Isolation of Human Lung Mast Cells (HLMC) was performed as described in our previous articles (13). HLMCs (10^6^ cells) were either left untreated (used as controls) or activated using the anti-FCεR1 antibody at 1:300 dilution (MAB6678, R&D Systems) for 24 h at 37°C and the supernatants were collected after centrifugation and frozen until later use.

### Treatment of ASM Cells With HLMC Conditioned Media

Treatment of ASM cells with HLMC conditioned media was performed as described previously (4, 5). The analysis of bronchial biopsies revealed a mean mast cell density in asthmatic ASM bundles of ~4 ASM cell:1 HLMC, therefore we treated serum-deprived ASM cells with different conditioned media at a dilution of 1:4 (25% v/v) including (i) FCεR1-activated mast cells, (ii) non-FCεR1 activated mast cells (control for mast cell activation) (iii) mast cell media (control for mast cell media) for 24 h at 37°C and 5% CO2. The next day, media were discarded and ASM cells were washed twice with ITS media before new ITS media was added to the ASM cells containing 10 ng/ml TNFα alone, or in the presence of fluticasone propionate (FP) (100 nM) and further incubated for 24 h at 37°C and 5% CO2. The supernatants were then collected and stored at −20°C for later use.

### ELISA

ELISA for the different chemokines was performed as described previously (14) with 50 μl cell supernatants using the R&D Systems DuoSet kits according to the manufacturer's instructions.

### RT2 Profiling PCR GC Signaling Array

Targeted transcriptomic analysis was performed as described in our previous study (15). We choose a PCR array that analyses the expression of a focused panel of 84 genes known to be induced by glucocorticoids. cDNA from healthy ASM cells was pre-amplified using the RT2 first strand cDNA kit, according to the manufacturer's instructions (SabBioscience, Qiagen). A RT2 profiler human Glucocorticoid signaling PCR array (PAHS-154Z) was used for quantitative PCR in the Strategene MX3000P system according to the manufacturer's instructions. Results were calculated using the 2^−Δ*ΔCt*^ method with normalization to two housekeeping genes (16).

### Quantitative PCR

Quantitative PCR was performed as described previously (17). Primers were GILZ forward: 5-TCTGCTTGGAGGGGATGTGG-3 and reverse: 5-ACTTGTGGGGATTCGGGAGC-3; MKP-1 forward: 5-GACGCTCCTCTCTCAGTCCAA-3 and reverse: 5-GGCGCTTTTCGAGGAAAAG-3; GAPDH forward: 5-TGCACCACCAACTGCTTAGC-3 and reverse: 5-GGCATGGACTGTGGTCATGAG-3; CCL5 forward: 5-AGTCGTCTTTGTCACCCGAA-3 and reverse: 5-TCCCAAGCTAGGACAAGAGCA-3; CXCL8 forward: ACTGAGAGTGATTGAGAGTGGAC and reverse: AACCCTCTGCACCCAGTTTTC CXCL10 forward: 5-GGATGGACCACACAGAGGCTGC-3 and reverse: 5-GCCCCTTGGGAGGATGGCAGT-3; FKPB5, TNFAIP and PIK3R1 primers were ordered from QIAGEN as proprietary information.

### Statistical Analysis

All data are presented as mean ± SEM. Statistical analysis was performed by two-way or one-way ANOVA with Bonferroni's correction for multiple comparisons. Differences were considered significant when *P* < 0.05. Statistical analysis was performed using GraphPad Prism 6 (GraphPad software, USA). For the RT^2^ profiler PCR array, student's *t*-test was applied and followed by 5% False Discovery Rate (FDR) with two-stage step-up procedure of Benjamini, Krieger, and Yekutieli.

## Results

### Effect of Activated and Non-activated Mast Cell Condition Media on TNF-α Induced Chemokine Expression in Healthy ASM Cells

**Figure 1:**
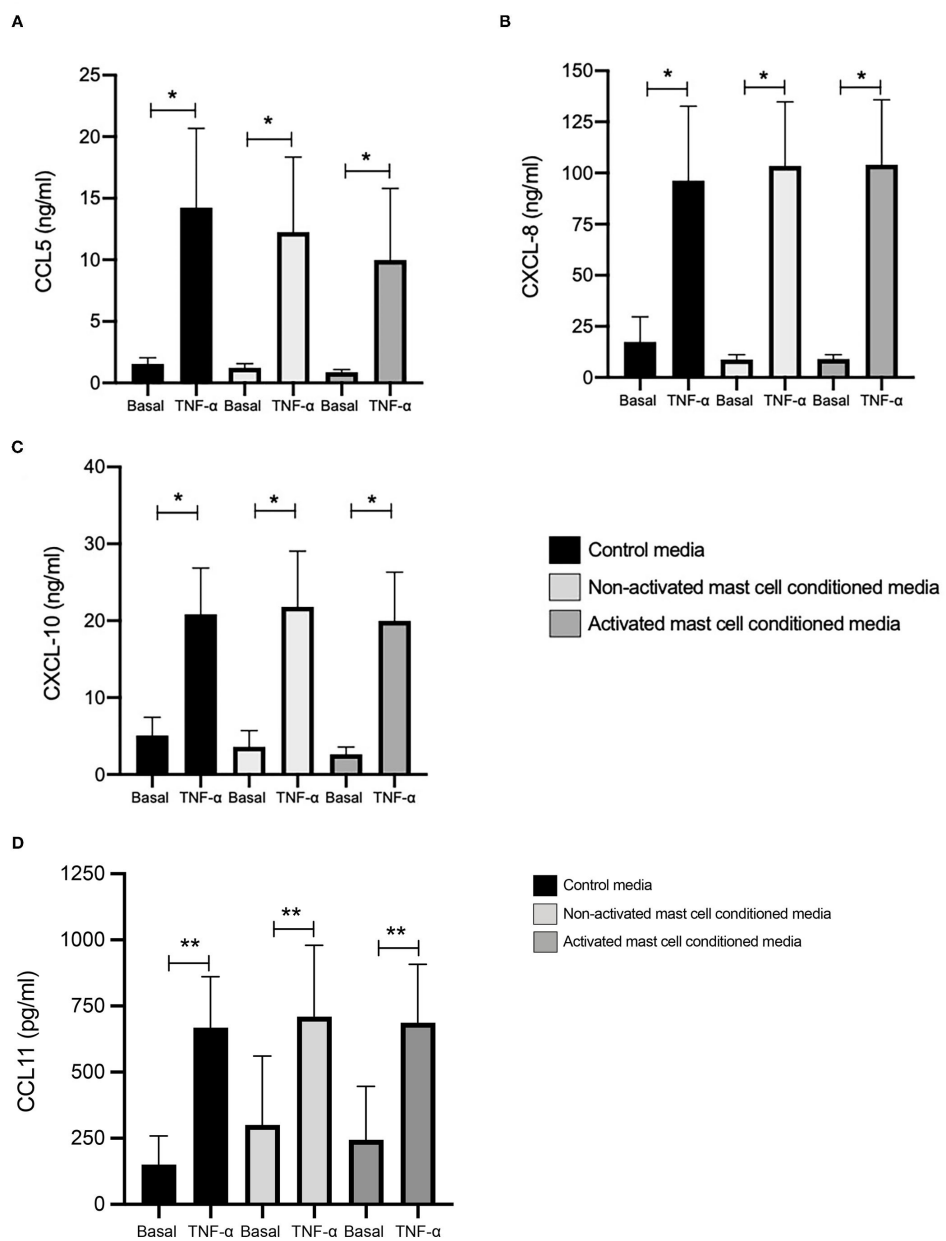
TNFα-induced chemokine production in ASM cells treated with control and 24-h mast cell conditioned media. Healthy ASM cells were pre-treated with control MC media, 24-h non-activated and activated mast cell conditioned media for 24 h. ASM cells were then washed and stimulated with TNF-α (10 ng/ml). The chemokine levels of CCL5 **(A)**, CXCL8 **(B)**, and CXCL10 **(C)** were assessed by ELISA. Data were presented as ng/ml of protein as Means ± SEM of *n* = 6 different cell lines. Comparisons between conditions were made using paired *t*-test (*p < 0.05).

We looked first at chemokine expression with and without TNF-α stimulation (10 ng/ml, 24 h) in healthy ASM primed with control media (as used for mast cell culture), and mast cell supernatants collected over 24 h (both non activated and FcεRI-activated). Levels of CCL5 at basal conditions were 1.07, 0.84, and 0.51 ng/ml, which were significantly increased by TNF-α to 12.22, 10.63, and 8.65 ng/ml in ASM primed with control (mast cell media), 24 h non-activated and activated mast cell supernatant, respectively (Figure 1A). Similarly, CXCL10 concentrations were 4.25, 3.04, and 2.07 ng/ml at basal levels, which were significantly increased by TNF-α to 17.51, 18.29, and 16.16 ng/ml, respectively. (Figure 1B) CXCL8 production at the basal levels was 14.91, 8.21, and 7.46 ng/ml which was induced significantly by TNF-α stimulation to 83.03, 88.83, and 89.99 ng/ml in healthy ASM primed with control (mast cell media), 24 h non-activated and activated mast cell supernatant, respectively (Figure 1C). Finally, concentrations of CCL11 were 150.4, 300.6, and 244.5 pg/ml at basal levels which were increased to 668.2, 710.2, and 686.9 pg/ml in the presence of TNF-α in ASM primed with control mast cell media, 24 h non-activated and activated mast cell supernatant, respectively (Figure 1D).

When we compared the net increase of the different chemokines induced by TNF-α, there was no significant difference between ASM primed with control (mast cell media), 24 h non-activated and activated mast cell supernatants, respectively. This suggests that activated or non-activated mast cell supernatant does not affect chemokine production in ASM cells induced by TNF-α.

### Effect of Activated and Non-activated Mast Cell Condition Media on Fluticasone Inhibition of TNF-α Induced Chemokine Protein Production

**Figure 2:**
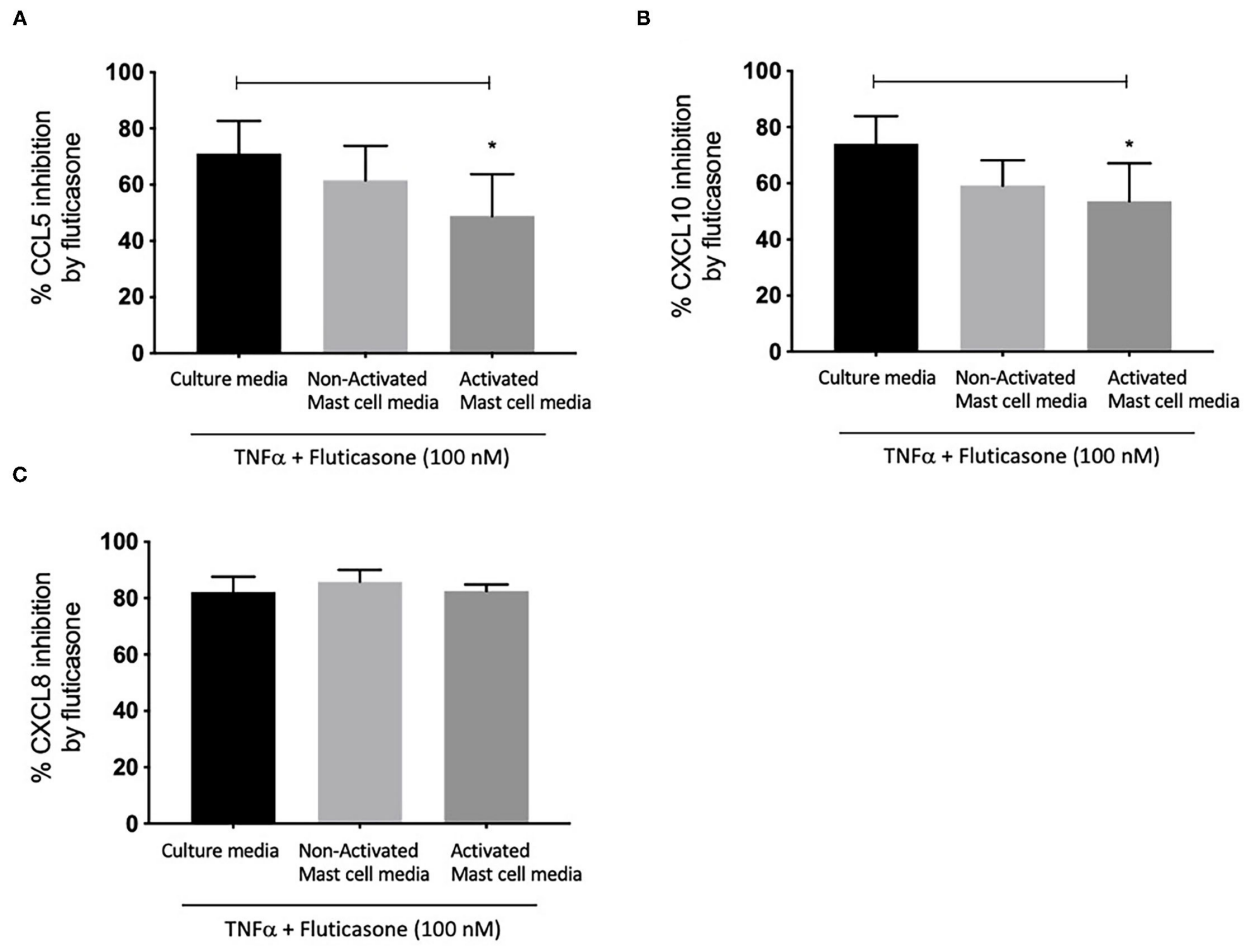
Inhibition of TNFα-induced-chemokines by fluticasone in ASM cells pre-treated with conditioned media from 24-h activated mast cells. Healthy ASM cells were pre-treated with control mast cells media, 24-h non-activated and activated mast cell conditioned media. ASM cells were then washed and stimulated with TNFα (10 ng/ml) with or without fluticasone (100 nM) for 24 h. Expression of CCL5 **(A)**, CXCL10 **(B)**, and CXCL8 **(C)** was assessed by ELISA with data presented as % of the chemokine responses in cells treated with TNFα alone (Means ± SEM of *n* = 6 different cell lines) (*p < 0.05). Comparisons between groups were made using one-way ANOVA and Tukey Test correction.

We next investigated whether conditioned media from activated and non-activated mast cells may modulate the anti-inflammatory action of corticosteroids by assessing the ability of fluticasone to inhibit chemokine production by TNF-α. Healthy ASM cells were primed with either control mast cell media, non-activated or activated mast cell-conditioned media for 24 h. This time point was selected to match our previous studies showing that a 24-h pre-treatment time with different cytokines [some of which are known to be produced by activated mast cells such as TNFα or IFNγ (18)] was able to alter corticosteroid sensitivity in ASM cells (19). *ASM cells* were then stimulated with 10 ng/ml TNF-α alone or in the presence of 100 nM fluticasone for 24 h, an experimental approach that allowed us to uncover the factors capable of affecting corticosteroid responsiveness in ASM cells (17). Fluticasone strongly inhibited TNF-α-induced-CCL5 production reaching a 71.1 ± 11.57% reduction in ASM cells pre-treated with control mast cell media. Interestingly, while the inhibitory action of fluticasone was not affected in cells primed with non-activated mast cell-conditioned media (61.61 ± 12.28% inhibition of CCL-5 production), incubating ASM cells with activated mast cell-conditioned media reduced fluticasone suppressive action to 48.84 ± 14.98% (*p* < 0.05) (22.27 ± 6.16% loss compared to the response in control media) (Figure 2A). Similarly, fluticasone reduced TNF-α-induced CXCL10 by 74.16 ± 9.76% in cells pre-treated with control mast cell media. However, fluticasone suppressive action was reduced to 53.65 ± 13.5% for CXCL10 in cells primed with activated mast cell-conditioned media. Furthermore, the action of fluticasone was not affected in ASM cells primed with non-activated mast cell conditioned media (Figures 2B,C).

Lastly, it was interesting to note that the suppressive action of fluticasone on TNFα-induced-CXCL8 was not affected in cells primed with either activated or non-activated mast cell conditioned media (85.84 ± 4.15% and 82.56 ± 2.28% inhibition, respectively) when compared to the inhibitory response of fluticasone seen in ASM cells primed with control mast cell media (82.25 ± 5.35%) (Figure 2C).

These studies show that conditioned media from activated mast cells can impair the anti-inflammatory action of fluticasone in ASM cells in a gene-specific manner.

### Effect of Activated and Non-activated Mast Cell Condition Media on Fluticasone Inhibition of TNF-α Induced Chemokine MRNA Expression

We next investigated whether mast cell-conditioned media affected the inhibitory action of fluticasone on chemokine expression at the transcriptional level. Hence, as before, ASM cells pre-treated with different mast cell-conditioned media were then stimulated with 10 ng/ml TNF-α alone or in the presence of 100 nM fluticasone for 6 h before mRNA isolation was carried out for qPCR analysis.

**Figure 3:**
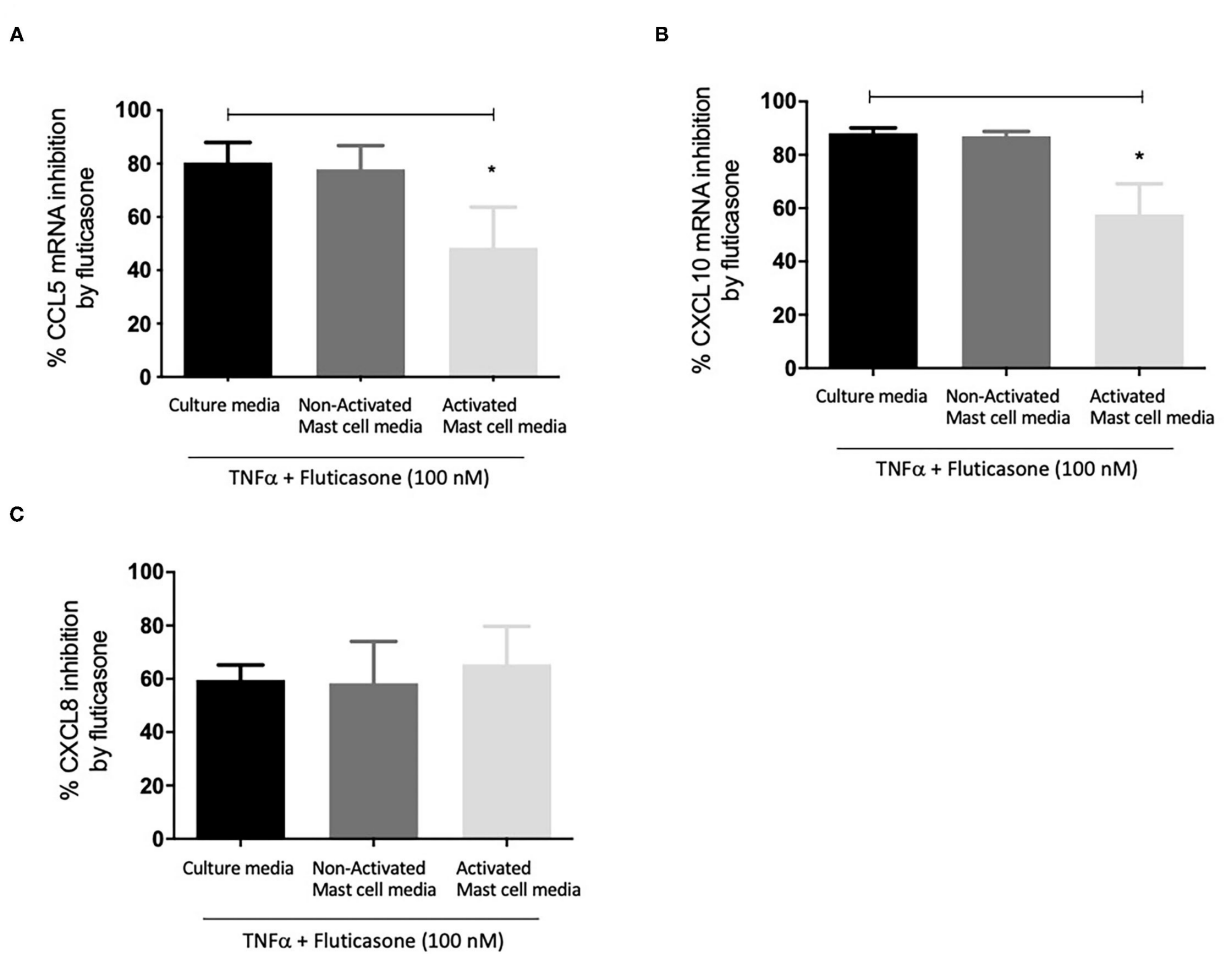
Inhibition of TNFα-induced chemokine mRNA expression by fluticasone in ASM cells pre-treated with conditioned media from 24-h activated mast cells. Healthy ASM cells were pre-treated with control mast cell media, conditioned media from 24-h non-activated, or 24-h activated mast cells for 24 h. ASM cells were then washed and stimulated with 10 ng/ml TNF-α with or without 100 nM fluticasone for 8 h. Chemokine expression of CCL5 **(A)**, CXCL10 **(B)**, and CXCL8 **(C)** was assessed by real-time PCR. Data are presented as % inhibition from chemokine responses in cells treated with TNFα alone (Means ± SEM of *n* = 5 different cell lines, *p < 0.05). Comparisons between groups were made using one-way ANOVA and Tukey Test correction.

Figure 3A shows fluticasone inhibited TNF-α-induced CCL5 mRNA expression by 80.47 ± 7.45% in healthy ASM cells primed with control, an effect that was not affected in cells pre-treated with 24 h non-activated mast cell-conditioned media (77.91 ± 8.84% inhibition) while priming ASM cells with conditioned media from activated mast cells dramatically reduced the inhibitory action of fluticasone to 48.46 ± 15.25% (*P* < 0.05). Activated mast cell conditioned media led to an overall 32.01% reduction in the fluticasone inhibitory action on CCL5 mRNA expression when compared to its effect in ASM cells pre-treated with control mast cell media.

Similarly, TNF-α-induced CXCL10 mRNA expression was inhibited by fluticasone by 88.15 ± 1.96% and 86.96 ± 1.86% in ASM cells primed with control mast cell media or 24 h non-activated mast cell conditioned media, respectively. In ASM cells first pre-treated with activated mast cell conditioned media, fluticasone inhibitory action was significantly reduced to 57.63 ± 11.5% (*P* < 0.05 vs. control mast cell media condition) (Figure 3B). Activated mast cell conditioned media led to an overall 30.52% reduction of fluticasone inhibitory action on CXCL-10 mRNA expression when compared to its effect in ASM cells pre-treated with control mast cell media.

Lastly, the inhibition of TNF-α-induced CXCL8 mRNA expression by fluticasone was not modulated by any of the mast cell conditions when compared to the response seen in cells treated with control mast cell media. Fluticasone led to 59.65 ± 5.57%, 58.36 ± 15.68% and 65.43 ± 14.27% reduction in TNF-α-induced CXCL8 mRNA expression in ASM cells that were primed with control mast cell media, non-activated or activated mast cell conditioned media, respectively (Figure 3C).

These data show that conditioned media from activated mast cells impair the anti-inflammatory action of fluticasone by reducing its ability to suppress gene expression at the transcriptional level.

### TGFβ Impaired the Ability of Fluticasone to Inhibit TNFα-Induced Gene Expression

**Figure 4:**
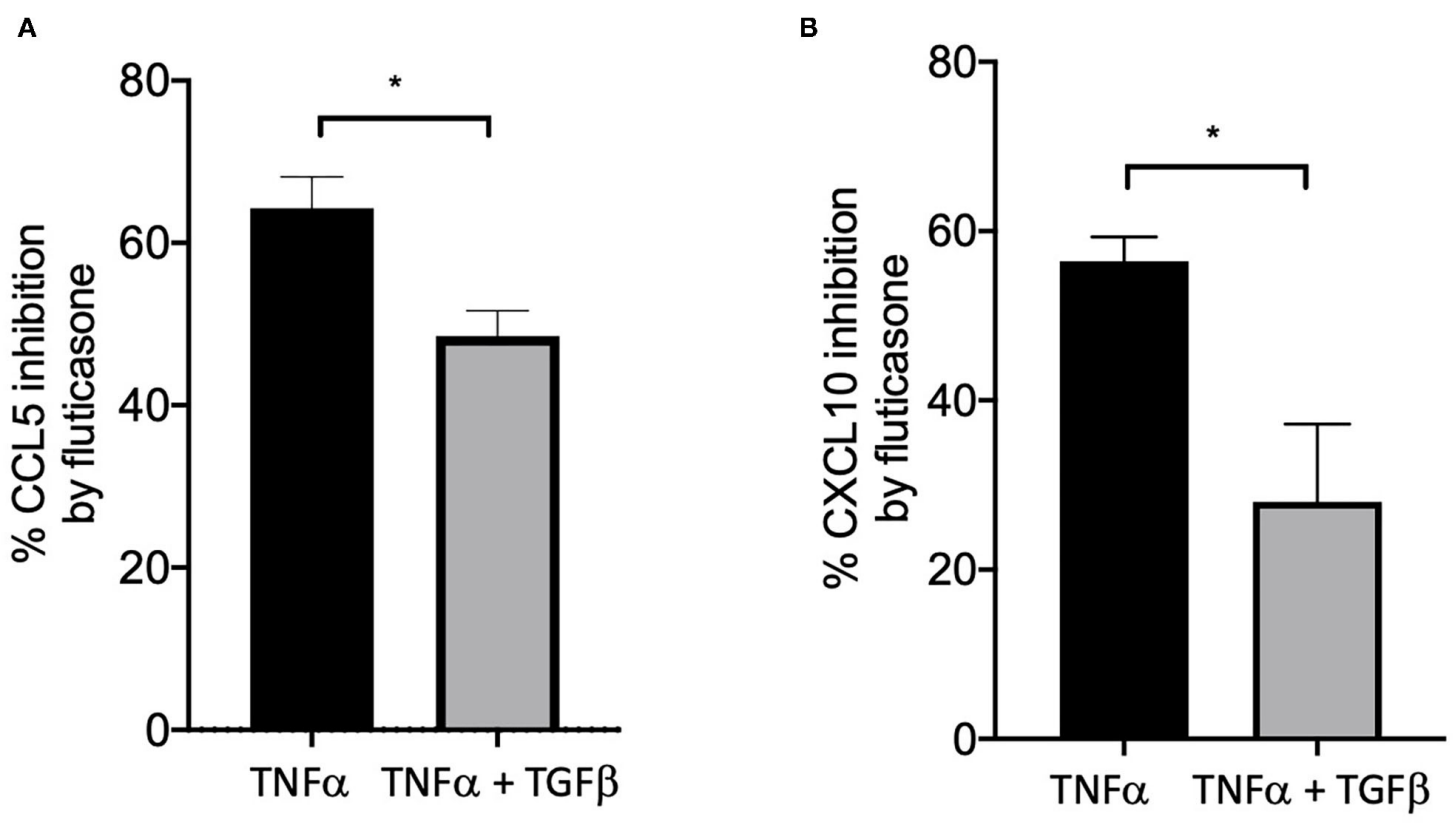
TGFβ impaired the capacity of fluticasone to inhibit TNFα-induced chemokine production. Healthy ASM cells were pre-treated with TGFβ (10 ng/ml) for 24 h before being stimulated with 10 ng/ml TNF-α with or without 100 nM fluticasone for 24 h. Chemokine expression of CCL5 **(A)** and CXCL10 **(B)** was assessed by ELISA with data presented as Means ± SEM of *n* = 3 different cell lines. Data are presented as % inhibition from chemokine responses in cells treated with TNFα alone or TNFα/TGFβ (Means ± SEM of *n* = 5 different cell lines, *p < 0.05). Comparisons between groups were made using paired student *t*-test.

We and others have shown that TGFβ was an important growth factor released by activated by mast cells (4) and capable of inducing corticosteroid resistance in lung epithelial cells (20). We found that in the presence of TGFβ, fluticasone inhibition of TNFα-induced chemokine production was reduced from 64.3 ± 3.9 to 48.5 ± 3% for CCL5 (Figure 4A) and from 56.4 ± 2.8 to 28 ± 9 (Figure 4B, *P* < 0.05), respectively. These data suggest a potential role of TGFβ in mediating corticosteroid insensitivity induced by mast cell conditioned media, although it is likely that other mediators may also be involved.

### Effect of Activated Mast Cell Conditioned Media on Fluticasone-Induced Gene Expression

**Figure 5:**
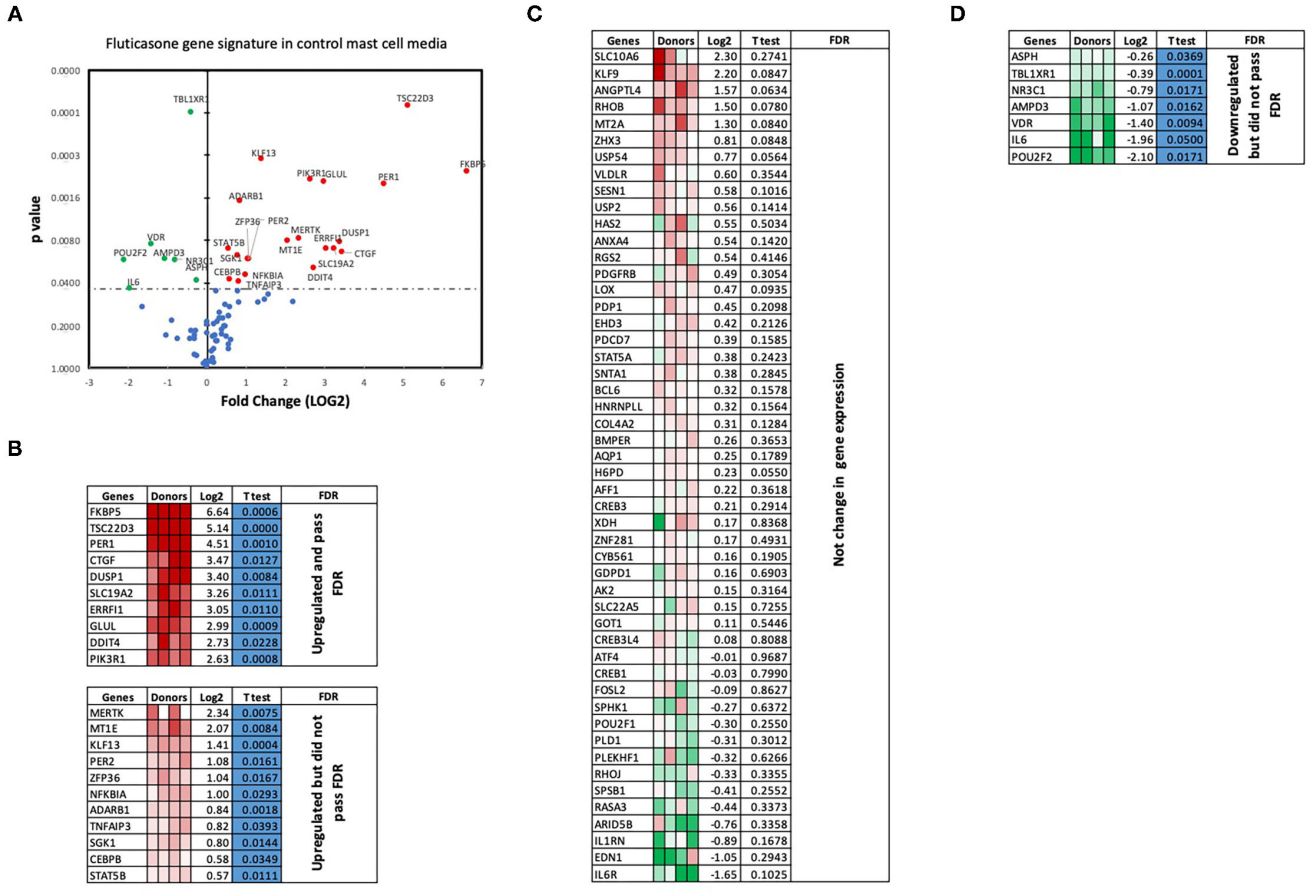
Volcano plot and heatmap of fluticasone inducible genes in ASM cells detected using the PCR array in cells pre-treated with control media. Data are presented as statistical significance (*p*-value) vs. fold change (log2 fold) on the y-axis and x-axis, respectively, in response to 100 nM fluticasone for 6 h in ASM cells first pre-treated with control mast cell media **(A)**. All genes were normalized to housekeeping genes (GAPDH and beta-actin and presented as fold Change (2^−Δ*ΔCt*^, log2 fold). **(B)** Genes upregulated that did pass or did not pass FDR, **(C)** genes with no expression change, and **(D)** genes that were downregulated but did not pass FDR. Student's *t*-test was used for statistical significance. All the data (*p*-value) were then adjusted using the False Discovery Rate approach (FDR) with a cut off *P* < 0.05.

**Figure 6:**
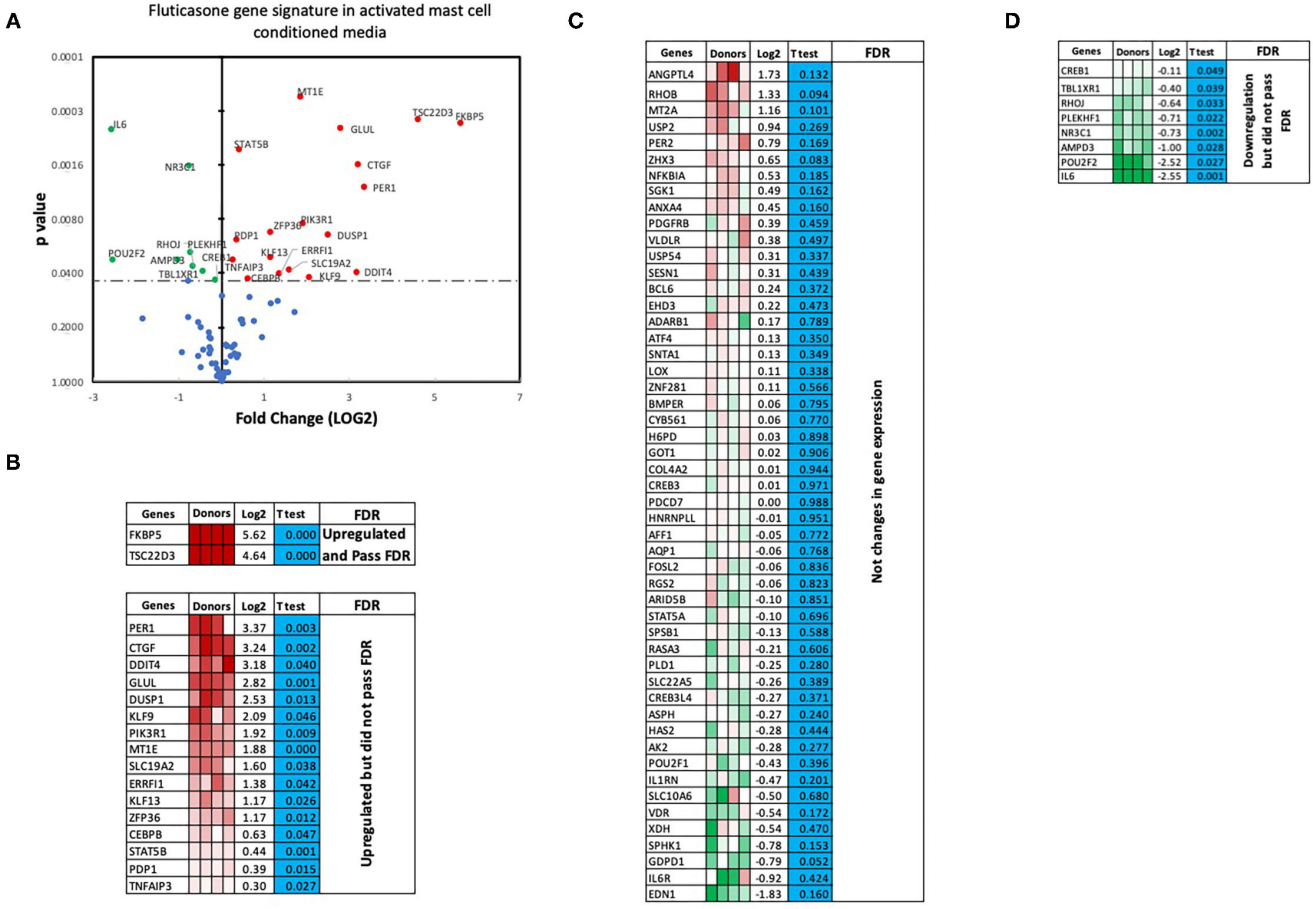
Volcano plot and heatmap of fluticasone inducible genes in ASM cells detected using the PCR array in cells pre-treated with activated mast cell conditioned media. Data are presented as statistical significance (*p*-value) vs. fold change (log2 fold) on the y-axis and x-axis, respectively, in response to 100 nM fluticasone for 6 h in ASM cells first pre-treated with control mast cell media **(A)**. All genes were normalized to housekeeping genes (GAPDH and beta-actin and presented as fold Change (2^−Δ*ΔCt*^, log2 fold). **(B)** Genes upregulated that did pass or did not pass FDR, **(C)** genes with no expression change, and **(D)** genes that were downregulated but did not pass FDR. Student's *t*-test was used for statistical significance. All the data (*p*-value) were then adjusted using the False Discovery Rate approach (FDR) with a cut off *P* < 0.05.

We next performed RT2 Profiler PCR Arrays focused on 84 key glucocorticoid inducible genes (Qiagen) to determine whether mast cell-conditioned media affected the fluticasone response by altering its transactivation properties. As shown in Figures 5A,B, fluticasone was able to induce the significant expression of a number of different genes with a log2 fold change ranging from 2.63 to 6.64 for the 10/84 genes which passed the false discovery rate (FDR). The genes that were significantly induced included FKBP5, TSC22D3, PER1, CTGF, DUSP1, SLC19A2, ERRFI1, GLUL, DDIT4 and PIK3R1. Some genes were either upregulated (Figure 5B) or down-regulated (Figure 5D) but none passed the false discovery rate (FDR). Fluticasone-dependent gene expression was significantly impaired in ASM cells pretreated with conditioned media from activated mast cells (Figures 6A,B) with only two genes (FKBP5 and TSC22D3) then passing the false discovery rate (FDR) with log2 fold changes of 5.62 and 4.64, respectively. The vast majority of genes induced by fluticasone in the presence of mast cell conditioned media did not change from baseline (Figure 5C) or were either upregulated (Figure 6B) or down-regulated (Figure 6D) but none passed the false discovery rate (FDR).

**Figure 7:**
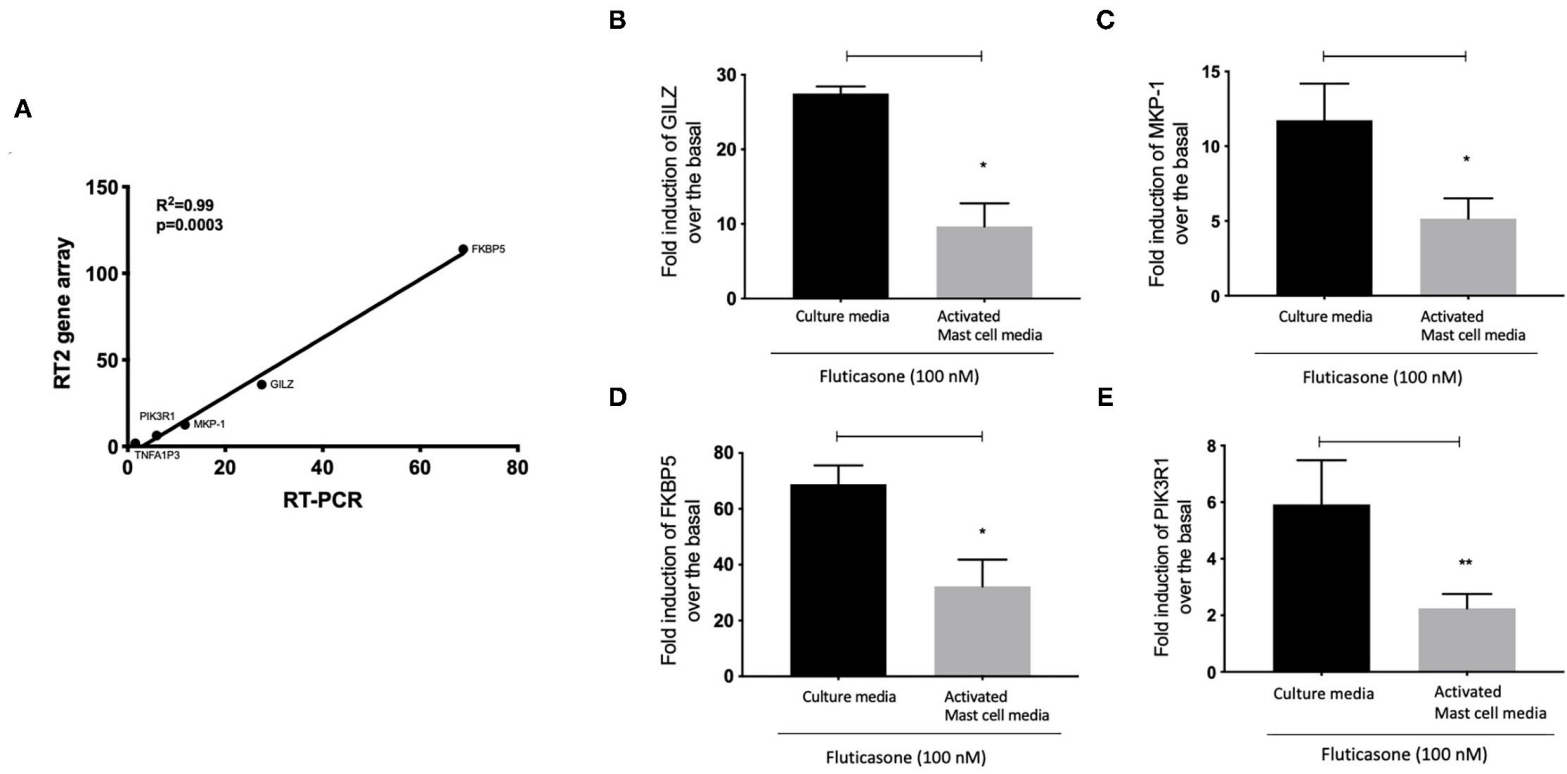
Individual real time PCR analysis confirmed the inhibition of fluticasone-induced gene expression by activated mast cell conditioned media. Healthy ASM cells were pre-treated with control mast cell media, conditioned media from 24-h non-activated, or 24-h activated mast cells for 24 h before cells were washed and stimulated with or without 100 nM fluticasone for an additional 6 h. Expression of anti-inflammatory genes of GILZ **(A)**, MKP-1 **(B)**, FKBP5 **(C)**, and PIK3R1 **(D)** was assessed by real-time PCR. Data were normalized to the housekeeping gene (GAPDH) and presented as fold change over the basal condition using the 2^−Δ*Δct*^ method as Means ± SEM, *n* = 7 different cell lines (*p < 0.05, **p < 0.01). **(E)** Validation of the gene array data using individual PCR. The log2 fold induction of the 5 genes shows a strong correlation with the results found using Qiagen RT2 profiling gene array and qRT-PCR, (R^2^ = 0.99, *P* = 0.0003).

We next validated the 3RT2 profiling gene array using individual qRT-PCRs (Figure 7A). Both methods showed that four genes among the top 10 upregulated by RT2 profiling gene array (FKBP5, TSC22D3, DUSP1, PIK3R1) had equal equivalent induction levels by qRT-PCRs (Pearson correlation coefficient R^2^ = 0.99, *P* = 0.0003). We also used the RT-PCR data to demonstrate the inhibitory effect of mast cell-conditioned media on fluticasone-induced transactivation (Figure 5). We confirmed that the induction of TSC22D3 (also known as GILZ) (Figure 7B), DUSP1 (also known as MKP-1) (Figure 7C), FKBP5 (Figure 7D) and PIK3R1 (Figure 7E) by fluticasone in ASM cells treated with mast cell media were significantly reduced in cells pre-treated with conditioned media from activated mast cells.

## Discussion

One of the major issues faced by patients with severe asthma (5-10%) is their poor response to the current asthma management guideline therapies that include GCs (21). The underlying mechanisms driving this poor response to GCs have not been identified although defects in the cellular response to GCs in both immune (22) and lung structural cells (23) have been proposed. The link between mast cells and a poor response to asthma medication can be implied from studies showing that omalizumab therapy can improve not only asthma symptoms (and pulmonary function) but also reduce the need for high dose inhaled or oral GCs (24). Our present study demonstrates that activated mast cells can blunt the anti-inflammatory action of GC in human ASM cells, a key dysfunctional component of the airway wall in asthma.

Our conditioned media approach revealed that mediators released from FCεR1-activated mast cells can alter the anti-inflammatory action of GC, as no effect was seen in ASM cells treated with culture media or conditioned media from resting (non-activated) mast cells. Indeed, we found that the repression of TNFα-induced protein and mRNA expression of CLL5 and CXCL-10 expression by fluticasone (~70-80% inhibition) could be significantly reduced in cells following a 24-h incubation with conditioned-media from FcεRI-activated mast cells (Figure 2). Our previous study, using a similar experimental approach, demonstrated that media from activated mast cells (but not from non-activated mast cell media) could lead to a drastic reduction of β2-adrenoceptor function in ASM cells as a result of receptor phosphorylation on tyrosine residues (4). We and others have also confirmed that mast cell mediators can affect multiple responses in ASM cells including migration (25), contractility (26, 27) and cytokine production (28, 29). Although the nature of the mediators capable of altering ASM response to GC in our study has not been investigated, a number of cytokines (TNFα, IFNγ, IL-17, IL-4, IL-13, macrophage migration inhibitory factor), alarmins (TSLP), growth factors (TGFβ) produced by activated mast cells have been shown to dampen GC response when tested individually in other asthma relevant cells [reviewed in (3)]. Among those, IL-4, IL-13, IL-17, TNFα, IFNγ, TSLP, TGFβ have been reported to possess functional receptors on ASM cells raising the possibility that these cytokines, most likely acting in concert, could be involved in reducing GC responsiveness induced by activated mast cell media. Indeed, we showed for example that a combination *of* TNFα/IFNγ *was capable* of inducing GC insensitivity in healthy ASM cells (14, 19, 30). It is unlikely that TNFα/IFNγ is responsible for the loss of GC response induced by *activated* mast cell *media* as CXCL8 was also affected by this cytokine combination (Figure 2). We now show exogenous TGFβ had the capacity on its own to reduce fluticasone response (Figure 4), in line with recent studies identifying TGFβ as a novel player in mediating corticosteroid sensitivity through its action on airway epithelial cells (20). We have previously reported that TGFβ was among the top growth factors released by activated mast cells alongside GM-CSF, HGF, or IGF-II although many others were also increased but did not reach significance due to small sample size and/or sensitivity of the assay (4). Whether TGFβ alone plays a critical role in mediating the reduced corticosteroid sensitivity driven by mast cell conditioned media is unlikely for various reasons. Exogenous TGFβ affected corticosteroid responses in ASM cells at >10 ng/ml, a concentration which is significantly greater than levels usually being produced by activated mast cells [pg-low ng/ml range (27)]. In addition, low concentrations of TGFβ (0.5-1 ng/ml range) have been described to effectively trigger various cellular responses in ASM cells including cell proliferation (31), and modulation of chemokine production such as eotaxin (32), or fractalkine (33). In epithelial cells, very low concentrations of TGFβ (4-100 pM) induced corticosteroid resistance (20). The ability of mast cells to produce multiple mediators that have the capacity to alter corticosteroid response (discussed above), raises the question of whether the impaired response to corticosteroids induced by mast cell conditioned media results from a combined action of multiple mediators. Rather than trying to tease out the possible ones involved (which will be a daunting task knowing the various mediators produced by mast cells), we tried instead to focus on the mechanisms by which mast cell-conditioned media altered GC response in ASM cells.

We found that while conditioned media from activated mast cells impaired the capacity of fluticasone to repress CCL5/CXCL10 production by TNFα, suppression of CXCL8 was still preserved. This may be explained, at least in part, by the fact that regulation of CXCL8 by fluticasone, but not that of CCL5 or CXCL10, involved mechanisms acting at both transcriptional and translational levels. Indeed, the magnitude of CXCL8 inhibition by fluticasone at the protein level was found to be significantly greater compared to that at the mRNA level (82.25 ± 5.35 vs. 59.65 ± 5.57% inhibition, *p* = 0.0174). In lung fibroblasts or airway epithelial cells, CXCL8 repression by dexamethasone was also reported to be complex and involve transcriptional regulation at multiple levels (34, 35). This suggests that the overall effect of mast cell conditioned media on the sensitivity of GCs may be gene-specific due to the nature of their anti-inflammatory mechanisms involved. The fact that CXCL8 remained a GC-responsive gene, despite the presence of conditioned media from activated mast cells, was an unexpected observation. High CXCL8 levels have been reported in treatment-refractory asthma patients (36–38), implying that mechanisms driving CXCL8 production may be insensitive to GC therapy. The apparent discrepancy about CXCL8 sensitivity to GCs between our *in vitro* data and these *in vivo* observations may be explained by different factors. First, the increased CXCL8 levels observed in some steroid-refractory patients have been usually reported in sputum, serum, and BAL fluids which could originate from multiple cellular sources, including the epithelium and other structural/infiltrated immune cells. Second, the role of mast cells in the pathogenesis of these patients where high levels of CXCL8 have been detected was not investigated. Assessing whether markers of mast cell activation correlate with steroid-refractory features, such as CXCL8 production, would answer this question. Third, we and others demonstrated that CXCL8 production by ASM cells obtained from severe asthmatics was indeed insensitive to GCs (when compared to cells from healthy/non-severe asthmatics) (17, 39, 40). Altogether, these different studies suggest that the development of GC resistant features in ASM cells may result from a combination of different mediators originating from mast cells and others pro-asthmatic triggers known to impact corticosteroid therapy (i.e., viruses, cytokines) (3) but also the overall GC anti-inflammatory mechanisms.

We made the observation that GC insensitivity present in ASM cells derived from severe asthma was also reported to be gene-specific with CCL5, IL-6 and CCL11 being resistant to dexamethasone (or fluticasone), while CXCL10 still being repressed by either GCs (17, 39, 41). These results suggest that mast cell mediators repressed some but not all anti-inflammatory mechanisms driven by the GC receptor (GRα) which involve two main mechanisms that include transactivation (i.e., ability of GCs to induce expression of anti-inflammatory proteins) to an transrepression (i.e., ability to interfere with the expression of pro-inflammatory mediators) (22). Transactivation was shown to play an essential role in the therapeutic action of GCs in various cell types including ASM cells (42), where induction of some anti-inflammatory proteins such as TSC22D3 (GILZ) and DUSP1 (MKP1) were reported to mediate the inhibitory actions of GCs (43–45). Our gene array approach revealed that out of 84 GC-inducible genes examined, 10 genes (FKBP5, TSC22D3, PER1, CTGF, DUSP1, SLC19A2, ERRFI1, GLUL, DDIT4 and PIK3R1) were significantly induced by fluticasone in cells treated with control conditions (all passed FDR cut off P < 0.05) (Figure 5B). Some of these GC-inducible genes were also reported in BEAS-2B cells treated with budesonide (ERRFI1, FKBP5, PER1, TSC22D3) (46), in ASM cells treated with dexamethasone (FKBP5, PERR1, PI3KR1, GLUL, TSC22D3) (47) or in lung epithelial cells treated with fluticasone, where FKBP5 and TSC22D3 were among the most highly induced genes (48). We made the interesting observation that only 2 out these 10 genes (namely FKBP5 and TSC22D3, albeit reduced) remained significantly up-regulated by fluticasone following treatment with activated mast cell-conditioned media (Figure 6B). Our real time PCR analysis allowed us to demonstrate a similar fold induction between array and qPCR data when looking at 5 randomly selected genes (R^2^ = 0.99, *p* = 0.0003, Figure 7A). The qPCR assays also confirmed that fluticasone-induced gene expression was indeed reduced by activated mast cell-conditioned media (Figures 7B-E), further supporting a defective GC-induced GRα transactivation property by activated mast cells. An impaired GRα transactivation was also reported as a mechanism for explaining the differential GC insensitivity seen in ASM cells from severe asthmatics (17). Increased expression level of the protein phosphatase 5 (PP5) driving a decreased GRα phosphorylation was previously described as the main underlying mechanism (17). Unfortunately, we failed to detect any changes in PP5 expression following treatment with activated mast cell-conditioned media arguing against a PP5-dependent defect in GRα phosphorylation. Future studies are required to determine how activated mast cells blunt GC transactivation responses in ASM cells and whether a similar effect is occurring in other lung cells.

This study supports the new concept that activation of mast cells in the lungs can decrease the anti-inflammatory action of GCs in ASM cells by affecting GRα transactivation. Because infiltration of mast cells within the ASM tissues is a defining feature of asthma, our data suggest that the persistence of GC-insensitive features in the lungs of severe asthmatics may result from their interaction with lung structural cells.

## Data Availability Statement

The raw data supporting the conclusions of this article will be made available by the authors, without undue reservation.

## Ethics Statement

The studies involving human participants were reviewed and approved by Leicestershire, Northamptonshire, and Rutland Research Ethics Committee (references 4977, 04/Q2502/74 and 08/H0406/189). The patients/participants provided their written informed consent to participate in this study.

## Author Contributions

AA, JH, FA, MB, AH, and LK performed the experiments and generated and analyzed the data. KMR and OT helped with the experiments and data analysis. YA with the help or PB conceived the project, designed the experiments, analyzed the data, and wrote the paper. All authors contributed to the article and approved the submitted version.

## Funding

This study was supported by the National Institute for Health Research Leicester Biomedical Research Centre Respiratory and funded in part by R01HL111541 (OT). The views expressed are those of the author(s) and not necessarily those of the NHS, the NIHR and by Department of Health.

## Author Disclaimer

The views expressed are those of the author(s) and not necessarily those of the NHS, the NIHR and the Department of Health.

## Conflict of Interest

The authors declare that the research was conducted in the absence of any commercial or financial relationships that could be construed as a potential conflict of interest.

## Publisher's Note

All claims expressed in this article are solely those of the authors and do not necessarily represent those of their affiliated organizations, or those of the publisher, the editors and the reviewers. Any product that may be evaluated in this article, or claim that may be made by its manufacturer, is not guaranteed or endorsed by the publisher.
